# Integrating Armchair, Bench, and Bedside Research for Behavioral Neurology and Neuropsychiatry: Editorial

**DOI:** 10.3390/biomedicines10122999

**Published:** 2022-11-22

**Authors:** Masaru Tanaka, Ágnes Szabó, László Vécsei

**Affiliations:** 1 ELKH-SZTE Neuroscience Research Group, Danube Neuroscience Research Laboratory, Eötvös Loránd Research Network, University of Szeged (ELKH-SZTE), Tisza Lajos krt. 113, H-6725 Szeged, Hungary; 2 Department of Neurology, Albert Szent-Györgyi Medical School, University of Szeged, Semmelweis u. 6, H-6725 Szeged, Hungary; 3 Doctoral School of Clinical Medicine, University of Szeged, Korányi Fasor 6, H-6720 Szeged, Hungary

“To learning much inclined, who went to see the Elephant (though all of them were blind) that each by observation might satisfy the mind”

—John Godfrey Saxe

—Tittha Sutta

Medical sciences have been steadily paving an exploratory path toward understanding the mechanisms of mental suffering, such as depression, anxiety, cognitive impairment, and pain. Such progress in contemporary research is certainly appreciable, and its tempo appears to be gaining increasing impetus. Indeed, state-of-the-art biotechnology, information science, and imaging techniques may help reveal novel findings in mental illnesses. It is no coincidence that this Special Issue, just titling common symptoms typical to not only psychiatric disorders but also common illnesses, collected 15 research papers, including seven preclinical studies, three clinical studies, one computational medicine, and four review articles covering interdisciplinary topics, including psychotherapy. This editorial introduces original research and review articles published in the Special Issue “Crosstalk between Depression, Anxiety, Dementia, and Chronic Pain: Comorbidity in Behavioral Neurology and Neuropsychiatry 2.0”, the second volume of the Special Issue “Crosstalk between Depression, Anxiety, and Dementia” [1]. We discuss ongoing projects to benefit from this current driving force in behavioral neurology and neuropsychiatry.

Recent advancements in neuroscience have enabled researchers to probe the brain in larger regions, at the cellular level, and with increased receptor specificity [2,3,4,5,6]. Research is focused on finding scientific frameworks for understanding the neuropathophysiology of mental illnesses, exploring the molecular regulation of higher-order neural circuits and neuropathological alterations, which may lead to prefrontal cortex (PFC) dysfunction, eliciting the symptoms of mental illnesses [7,8,9,10,11,12,13]. The deficit in control and motor inhibition [14,15,16], in motor imagery or in the suppression of ongoing action [17], or in emotion perception, reactivity, and regulation [18,19,20], which depend on aberrant neural activity in the PFC associated with serious impulsivity problems, are characterized in neuropsychiatric disorders. Furthermore, functional alterations in the PFC affect the memory and learning abilities of psychiatric and brain-damaged patients. This evidence suggests that PFC dysfunctions cause impairment of aversive learning and emotional memory circuits, which might be transversal across many psychiatric disorders in humans and neurologic patients [21,22,23].

Experimental medicine employs in vitro systems as well as a wide variety of organisms [24,25,26]. The data collected using laboratory animals have led to significant leaps in understanding the effects of endogenous neuropeptides, neurohormones, and metabolites [27,28,29,30,31]. The initial step in animal research is to engineer typical animal models representing a certain human disease. Animal models are an essential tool to bridge the knowledge of data- and hypothesis-driven benchwork and its application to clinical bedside management. Nevertheless, assessing the validity of an animal model remains the greatest challenge. Model validity is determined by construct, face, and predictive validities. Construct validity ensures that a disease phenotype in modeling animals is induced by the currently understood pathomechanism of a disease.

Gene manipulation is one of the most popular methods to construct animal models of Alzheimer’s disease (AD). The authors in this Special Issue employed transgenic mice models: amyloid precursor protein (APP) 23 transgenic mice that overexpress human APP with the Swedish mutation (KM670/671NL), triple-transgenic mice of AD (3xTg-AD) that harbor a *Psen1* PS1M146V mutation and the co-injected APPSwe and tauP301L transgenes (Tg(APPSwe,tauP301L)1Lfa), and the pyruvate dehydrogenase lipoamide kinase isozyme 1 K465E gene knock-in mice. Those models have good face validity, which ensures that the signs exhibited in the models resemble those of human diseases. Particularly, the 3xTg-AD model displays that the accumulation of tau and amyloid plaques in the brain increases with age.

Muntsant and Giménez-Llort revealed an increasing APP level in genetic load- and aging-dependent manners, correlating with cognitive impairment and anxiety-like behaviors [32]. Giménez-Llort and colleagues investigated the chronological and behavioral aging of APP23 transgenic mice. The survival curves were better in male than in female APP23 mice and wild types. Age-related differences were observed, and variables related to stress, thigmotaxis, frailty, and cognition were more prominent in male APP23 mice in 12-, 18-, and 24-month-old time points compared to those of the wild-type counterparts. Muntsant and colleagues investigated the genetic and aging interactions of 3xTg-AD [33]. The study revealed an increased APP level in a genetic load-dependent manner, convergent synaptophysin and choline acetyltransferase levels, cognitive impairment coupled with the activation of the hypophysis–pituitary–adrenal axis, anxiety-like behaviors elicited by genetic load, and systemic organ injuries, showing the presence of the genetic- and aging-dependent vulnerability and compensation in AD [34]. Castillo-Mariqueo and colleagues conducted longitudinal and cross-sectional studies to assess physical and behavioral variables in 3xTg-AD, concluding that the transgenes modify functional trainings, especially in survival, physical resistance, and motor learning [35]. Santana-Santana and colleagues studied the impact of gender and the pyruvate dehydrogenase lipoamide kinase isozyme 1 K465E knock-in gene on behaviors by comparing homozygous, heterozygous, and wild-type mice. The difference between gender and the transgenic mice was observed in an anxiogenic environment during the middle age of the mice. The male transgenic mice showed increased anxiety. The authors concluded that various negative emotional valence, such as anxiety, were elicited by the interaction of sex and PI3K/Akt signaling [36]. Furthermore, gene manipulation is applied to construct animal models of other neuropsychiatric pathogenesis, including neurodevelopmental disorders and the decreased resilience of neuroplasticity as a pathogenesis of neuropsychiatric disorders [37,38].

Surgical intervention can simulate vascular dementia, the second most common neurocognitive disorder. Employing bilateral carotid artery stenosis in mice, Lee and colleagues reported that pro-inflammatory cytokines, rho-associated protein kinase, and mRNA levels of blood–brain barrier-related tight junction proteins were decreased; smooth muscle alpha-actin positive vessels were increased, cortex cell rearrangement was decreased, and microtubule-associated protein-2-positive neural cells was decreased in the hippocampus, simulating the clinical pathology of vascular dementia [39]. Thus, this model ensures high face validity.

Environmental risk factors also generate a disease phenotype. The chronic social defeat stress model is an ethologically valid animal model that exhibits behavioral and physiological phenotypes such as anxiety- and depression-like behaviors and the downregulation of serotonergic gene expression. Smagin and colleagues investigated the chronic effects of lithium chloride on anxiety-like behavior and the expression of serotonergic genes in the midbrain raphe nuclei of mice. The chronic lithium chloride administration elicited anxiolytic- and anxiogenic-like behaviors and the higher expression of serotonergic genes in the midbrain raphe nuclei. The authors concluded the increased expression of serotonergic genes occurs with the activation of the serotonergic system and elevated anxiety [40].

Health resilience has drawn increasing attention to one of the etiological factors of illnesses from molecular to social levels. Less mitochondrial stress resilience, the disturbance of thiol homeostasis, and contemporary lifestyles may reportedly contribute to the pathogenesis of neurological and psychiatric diseases, multiple sclerosis, and mental illnesses, respectively [41].

The prevalence of hypertension in schizophrenia (SCZ) patients and psychosis-related disorders reaches nearly 40%. Spontaneously hypertensive rat is an animal model of SCZ. Correia and colleagues reported that antipsychotics haloperidol and clozapine increased total lipids and decreased phospholipids in spontaneously hypertensive rats [42]. The findings are in line with those of SCZ patients. Thus, the spontaneously hypertensive rat model possesses high predictive validity that ensures translational ability between animal models and human disease. However, SCZ develops from heterogenic insults in the early neurodevelopmental stage, confirming the presence of various subgroups of SCZ. Vitamin D deficiency is highly prevalent in patients with SCZ; however, little is known about how vitamin D deficiency affects the disease course of SCZ, especially cognitive function. Gaebler and colleagues assess the correlation between the serum 25-OH-vitamin D levels, anticholinergic drug exposure, and neurocognitive functions in patients with SCZ, reporting a positive correlation of vitamin D levels with cognitive processing speed and a negative correlation of vitamin D levels of anticholinergic drug exposure [43]. Cognitive function, including memory and learning, is sustained by synaptic plasticity, which governs the fine-tuning of the synaptic strength and efficacy of the synaptic transmission. Carnosine is an endogenous anti-aging dipeptide, highly concentrated in brain and muscle tissues. Caruso and colleagues conducted a systematic review and a meta-analysis, concluding that a dose of 500 mg–1 g/day carnosine/anserine for 12 weeks improved cognitive function and verbal memory but not depressive symptoms [44].

Deep brain stimulation is a surgical procedure used to treat Parkinson’s disease, essential tremor, epilepsy, and dystonia, which is reportedly beneficial for treatment-resistant depression. Vila-Merkle and colleagues studied the therapeutic mechanisms of deep brain stimulation of the infralimbic cortex by electrophysiological recordings in the β-carboline FG 7142-induced anxiety model. The model exhibits the predominance of certain frequency bands during the anxiogenic state and the activation of subnetworks with specific oscillatory patterns. The study reported that the deep brain stimulation of the infralimbic cortex reversed the oscillatory pattern, restoring the communication of the amygdala–hippocampal network [45].

An increasing number of studies have focused on the fact that the tryptophan (Trp)–kynurenine (KYN) metabolic system not only plays a role in the pathogenesis of diseases but also serves as a potential biomarker for environmental health [46,47]. The Trp–KYN metabolic system refers to a group of endogenous bioactive metabolites arising via the KYN metabolic pathway from an essential amino acid Trp. The metabolic system adds more implications of their versatile biological activities than the traditionally used term, the Trp–KYN pathway that produces a group of simply categorizing neurotoxic and neuroprotectant KYN molecules. Indeed, KYN metabolites exhibit a wide range of bioactive properties, frequently showing a Janus-like face depending on their concentration and environment and possibly influencing the bioenergetic resilience of mitochondria [41,48]. Tanaka and colleagues featured the Trp–KYN metabolic system with special emphasis on its interaction with the immune system, including the tolerogenic shift towards chronic low-grade inflammation, to explore the linkage between chronic low-grade inflammation, KYN metabolites, and major psychiatric disorders, including depressive disorder, bipolar disorder, substance use disorder, post-traumatic stress disorder, SCZ, and autism spectrum disorder [49,50,51]. In parallel, the role of diet in maintaining mental health has been explored [52]. Furthermore, the link between air pollution and the pathogenesis of depression has been proven [53]. Novel therapeutics for neuropsychiatric conditions are under extensive study [54,55].

Furthermore, the Trp–KYN metabolic system and its metabolites have been discussed as a target of potential therapeutic molecules for cognitive impairment as well as for headaches [56,57]. Tanaka and colleagues discussed the involvement of the Trp–KYN system in chronic pain, addressing the components of the pain pathway, the components-based pain mechanisms, and central and peripheral pain sensitization arising from psychosocial and behavioral factors, which have been in a discounted trend in contemporary clinical nosology [50]. Pharmacotherapy and surgical intervention have their limits due to the development of drug intolerance and contraindications. Balogh and colleagues highlighted evidence in philosophically-rich interpretations and counseling techniques of existential–phenomenological psychotherapy and meaning-centered counseling techniques, and reviewed its effectiveness in the negative-emotion-management of terminally ill patients. The authors concluded that phenomenological psychotherapy might potentially play a synergistic role with the currently prevailing medication-based approaches for treating depression and anxiety [58].

Myalgic encephalomyelitis/chronic fatigue syndrome is a fatiguing medical condition caused by heterogeneous pathogenesis; thus, it is a challenging task to subgroup for personalized treatment. Simonato and colleagues revealed that interleukin-17A, fatty acid-binding protein 2, and 3-hydroxykynurenine were higher. However, KYN and serotonin were lower in myalgic encephalomyelitis/chronic fatigue syndrome patients, concluding that the clinical traits and serum biomarkers associated with inflammation, intestinal function, and Trp metabolism deserve to be explored for the development of personalized treatment [59].

Indeed, “personalized medicine” indicates “precision medicine”, which focuses on identifying an effective therapeutic approach based on the patient’s phenotypes. Artificial intelligence leverages computation and inference to augment logical insights into artificial intelligence, enabling the system to reason and learn, thus potentially empowering clinical decision-making. The Research Domain Criteria Initiative attempts to reconceptualize mental disorders with multidimensional data. Komatsu and colleagues reviewed the application of economic and machine learning frameworks to neuroscience to present neural mechanisms of cognitive processes, such as decision-making, the translation of machine learning approaches to clinical sciences, and the identification of functional connectivity as disease classifiers for schizophrenia, bipolar disorder, depression, anxiety disorders, and autism spectrum disorder. The authors proposed artificial intelligence algorisms as tools for precision psychiatry, which potentially surpass the International Classification of Diseases and Diagnostic and Statistical Manual of Mental Disorders [60].

Clinical, experimental, and computational medicines have witnessed notable advances in understanding pathogenesis, making precise diagnoses, and exploring novel treatments for neuropsychiatric disorders. Research Domain Criteria is an ongoing initiative that allows a multidimensional and intersectional approach to link mental illnesses, as extreme conditions that deviate from healthy norms, to genomic, neuroscience, and behavioral sciences across reified International Classification of Diseases and Diagnostic 11 and Statistical Manual of Mental Disorders 5. Thus, the endeavor potentially provides an alternative taxonomy that complements the current system. Meanwhile, computational medicine seeks to advance healthcare by analyzing, modeling, simulating, and visualizing biological systems and medical conditions in a virtual environment in an attempt to improve preventive, diagnostic, prognostic, predictive, and therapeutic measures. Such in silico approaches employ artificial intelligence, machine learning, and deep learning to analyze patient datasets and bioinformatics databases, apply computational algorithms, and utilize big data analytics tools at molecular, cellular, and organism levels. Accordingly, science and medicine have been unintermittingly advancing, firmly reinforcing the interdisciplinary translatability and synthesizability of individual research, and thus hopefully leading to a future paradigm shift in diseases and medicine.

The blind never see the elephant, but together they might attain a closer image.

“the Elephant is very like a wall, spear, a snake, a tree, fan, rope”

“Though each was partly in the right, Additionally, all were in the wrong!”

“Additionally, prate about an Elephant Not one of them has seen!”

—in memory of Dr. Mutsuo Shimizu

## Abbreviations

3xTg-AD	triple-transgenic mice of Alzheimer’s disease
AD	Alzheimer’s disease
APP	amyloid precursor protein
KYN	kynurenine
PFC	prefrontal cortex
SCZ	schizophrenia
Trp	tryptophan
